# Rare Neuroendocrine Tumor of the Bladder: A Case Report

**DOI:** 10.7759/cureus.92132

**Published:** 2025-09-12

**Authors:** Laritza Fernandez-Claro, Manuel Pizarro-Mondesir, Francisco Baralt-Nazario, Omar E Soto-Aviles

**Affiliations:** 1 Urology, Universidad Central del Caribe School of Medicine, Bayamon, PRI; 2 Urology, University of Puerto Rico, Medical Sciences Campus, San Juan, PRI; 3 Urology, Bayamon Medical Center, Bayamon, PRI

**Keywords:** bladder ca, neuroendocrine tumor of the bladder, neuroendocrin tumor, urinary bladder ca, well-differentiated neuroendocrine tumor of the bladder

## Abstract

Well-differentiated neuroendocrine tumors (WD-NETs) of the bladder are notably rare, representing only a small subset of neuroendocrine malignancies in this organ. We present a case of an asymptomatic 67-year-old Hispanic male in whom a WD-NET was incidentally identified during routine surveillance cystoscopy, six months following anterior urethroplasty with a buccal mucosal graft. The patient underwent two transurethral resections of the bladder tumor (TURBT). This case highlights the diagnostic challenges associated with WD-NETs given their rarity and reinforces the need for clinical awareness, even in patients without typical risk factors or symptoms.

## Introduction

Urothelial cancer is the sixth most common cancer in men and the 17th in women, with bladder cancer (BCa) being the most prevalent type, comprising 90% of all urothelial cancers [1]. Neuroendocrine tumors (NETs) make up just 1% to 2% of all genitourinary cancers [2]. NETs more commonly develop in the gastrointestinal (GI) tract and lungs. Less commonly, they can develop in the kidney, ureter, renal pelvis, and bladder (<1%) [2]. The 2022 World Health Organization (WHO) classification of Urinary System and Male Genital Organs divided NETs into four categories: well-differentiated neuroendocrine tumors (WD-NETs), small cell neuroendocrine carcinoma, large cell neuroendocrine carcinoma, and paragangliomas [3]. Small cell neuroendocrine carcinoma represents the most prevalent subtype of NETs in the bladder, though it remains exceedingly rare, with an estimated annual incidence of 0.14 cases per 100,000 population [3]. In contrast, large cell neuroendocrine carcinoma and WD-NETs of the bladder are notably rare, accounting for only a small subset of neuroendocrine malignancies in the bladder. Around 30 to 40 cases have been recorded in previous literature [3].

WD-NETs of the bladder may usually present with microscopic or gross hematuria. On cystoscopy, they are more commonly found in the bladder trigone or neck regions, typically presenting as nodular or polypoid masses smaller than 1 cm [4]. Pathology findings often show monotonous cells with rounded nuclei, stippled “salt and pepper” granular chromatin, and eosinophilic cytoplasm arranged in nested, pseudoglandular, or trabecular patterns. A unique feature of bladder WD-NETs is the presence of eosinophilic Paneth-like cytoplasmic granules [4-6].

Non-urothelial BCa are diagnosed, evaluated, and staged using methods similar to urothelial BCa [7]. Due to their rarity, treatment is primarily guided by retrospective studies and limited prospective data, as randomized trials are challenging to perform [7]. Surgery remains the primary treatment, often combined with chemotherapy or radiation in select cases [7]. WD-NETs are generally low-grade, confined to the bladder, and tend to have a favorable prognosis. However, there are reported case reports showing WE-NETs of the bladder that present with regional or distant metastasis [7,8]. Consequently, treatment alternatives should be done on a case-by-case basis. This case report presents a case of NET of the bladder in a patient who presented with no symptoms and was diagnosed during surveillance cystoscopy after urethroplasty with buccal mucosal graft.

## Case presentation

**Figure 1:**
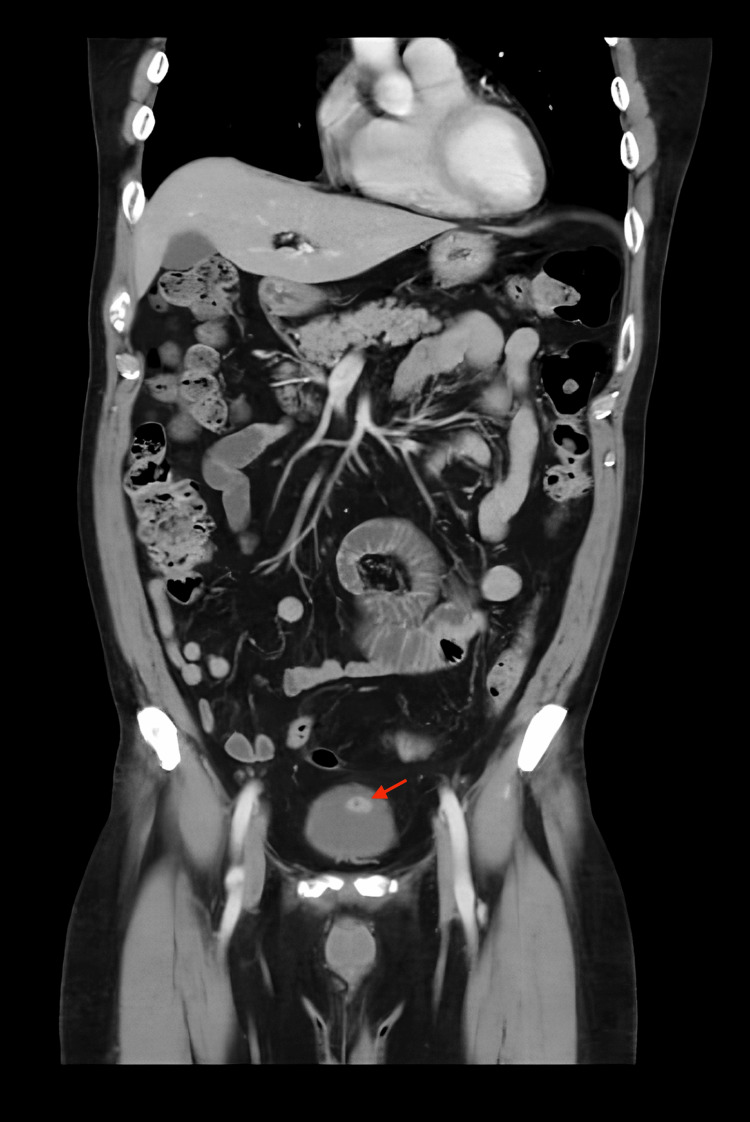
Contrast coronal abdomenipelvic CT scan demonstrating an enhancing mural nodule on the anterior bladder wall suggestive of malignancy. The red arrow in the image points to an enhancing mural nodule on the anterior bladder wall measuring 1.0 × 1.7 × 1.0 cm, with prominent feeding vessels arising from the left inferior vesical artery.

**Figure 2:**
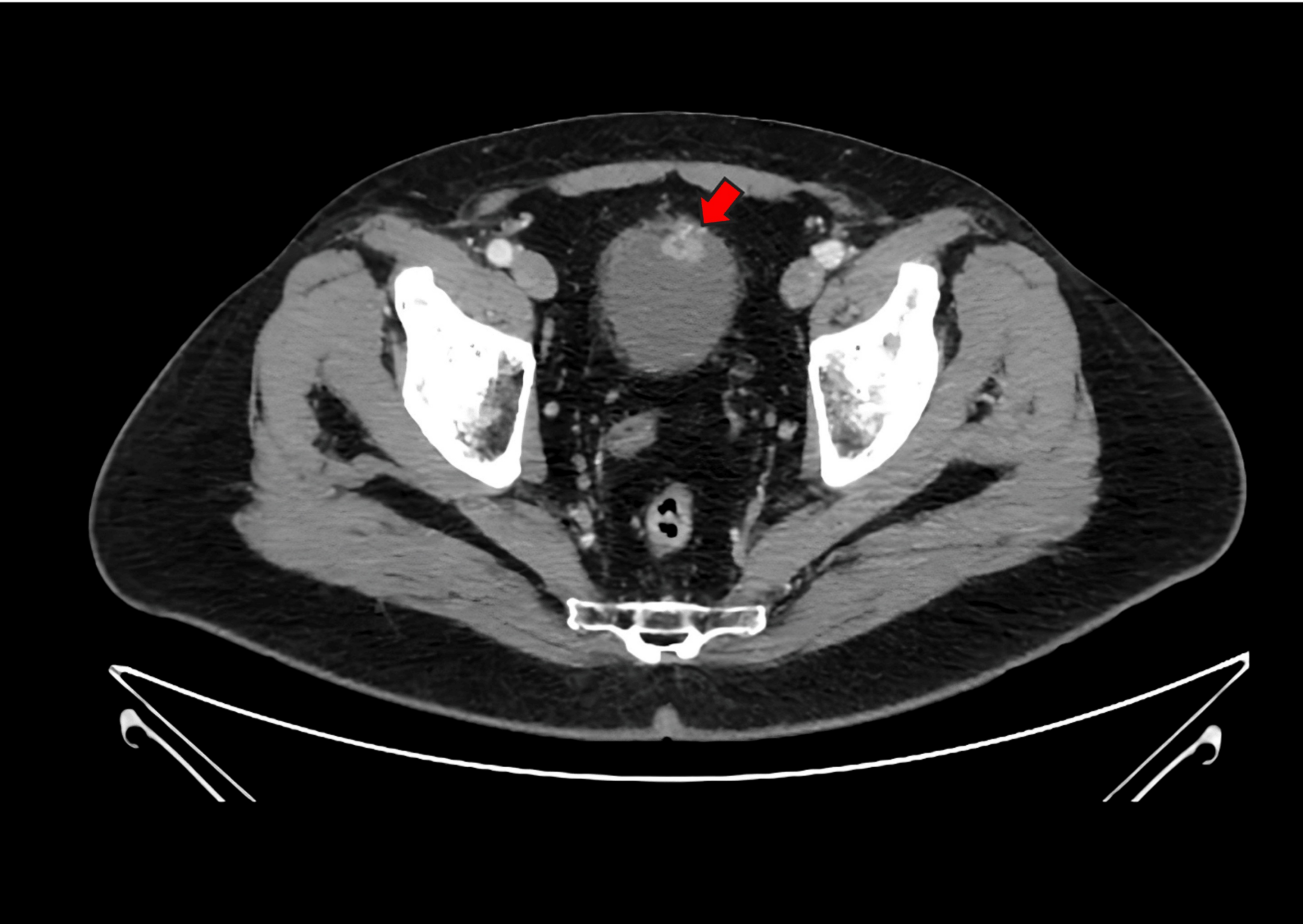
Contrast axial abdomenipelvic CT scan demonstrating an enhancing mural nodule on the anterior bladder wall suggestive of malignancy. The red arrow in the image points to an enhancing mural nodule on the anterior bladder wall measuring 1.0 × 1.7 × 1.0 cm, with prominent feeding vessels arising from the left inferior vesical artery.

A 67-year-old Hispanic male was evaluated for a bladder tumor incidentally identified during surveillance cystoscopy, performed six months following anterior urethroplasty with a buccal mucosal graft. The patient had a past medical history of urethral stricture, hyperlipidemia, hypertension, and arthritis. He had no previous smoking history. His surgical history includes anterior urethroplasty with buccal mucosal graft, direct vision internal urethrotomy, septoplasty, and rotator cuff repair. He had no previous family history of cancer or genetic conditions. The patient denied gross hematuria, prior microscopic hematuria on urinalysis, or any other urinary symptoms. Six-month post-urethroplasty surveillance cystoscopy was remarkable for an anterior bladder wall tumor measuring 3 cm. The remainder of the bladder demonstrated normal contour, with no evidence of additional tumors or suspicious lesions. The prostate was nonobstructive, and the urethra showed a well-healed and patent graft. Preoperative abdominopelvic CT scan revealed an enhancing mural nodule on the anterior bladder wall measuring 1.0 × 1.7 × 1.0 cm, with prominent feeding vessels originating from the left inferior vesical artery (Figures 1-2).

The patient was subsequently taken to the operating room for transurethral resection of the bladder tumor (TURBT). Rigid cystoscopy showed a 3 cm bladder tumor with no other suspicious lesions. The tumor was resected completely and sent to pathology. Pathology results showed a WD-NET with unremarkable muscularis propria. Immunohistochemical analysis was then performed, revealing cellular expression of CD56, synaptophysin, chromogranin A, S-100, and GATA-3. No other cellular expression of tumor markers was identified. Finally, the Ki-67 proliferation rate was less than 1%, confirming a WD-NET. 

Following the TURBT, the patient underwent repeat MRI imaging, which demonstrated no evidence of residual tumor, recurrence, or metastasis in the pelvis. A postsurgical defect was noted at the mid-left anterior bladder wall. According to the American Urological Association (AUA) guidelines, in patients with variant histologies, such as NETs, a repeat TURBT should be performed when bladder-preserving treatment is being considered to accurately assess the clinical stage of the disease [7]. Following these guidelines, the patient underwent a second TURBT three months later. Cystoscopy showed no evidence of bladder tumor recurrence; however, the scar from the previous procedure was noted, resected, and sent to pathology. The pathology reports showed no evidence of recurrence of the neuroendocrine bladder tumor. The patient now continues close follow-up with future surveillance cystoscopy planned to monitor for bladder tumor recurrence and to assess urethral stricture disease.

## Discussion

NETs of the urinary bladder are rare, with an estimated annual incidence of fewer than one to nine cases per million [8]. It is most commonly diagnosed at an advanced stage and predominantly affects Caucasian males in their sixth to seventh decade of life [9]. Key risk factors include tobacco use and occupational exposure to carcinogens, such as industrial dyes. Patients typically present with hematuria, dysuria, and lower urinary tract symptoms (LUTS) [10]. 

NETs are a diverse group of neoplasms that originate from cells with both neural and endocrine features, capable of producing peptides and neuroamines [11]. NETs are classified based on their degree of differentiation (well vs. poorly differentiated) and proliferation index (Ki-67), which are critical for predicting behavior and guiding treatment [4,12]. WD-NET tumors, categorized as grade 1 (low) or grade 2 (intermediate), generally have a better prognosis. Conversely, grade 3 (high-grade) tumors, which are poorly differentiated, often show a high mitotic count and/or increased Ki-67 index (>5%), indicating a more aggressive clinical course [8,13]. In our case, the Ki-67 proliferation rate was less than 1%, confirming low-grade and better prognosis. 

Histologically, WD-NETs are composed of monotonous small cells with round to oval nuclei, finely stippled ("salt and pepper") chromatin, inconspicuous nucleoli, and moderate amounts of eosinophilic cytoplasm [4-6]. The tumor cells are typically arranged in trabecular, nested, pseudoglandular, or acinar patterns, often embedded in a richly vascular stroma [4]. Mitotic figures are rare, and necrosis is typically absent [5,6,12]. One of the hallmark features aiding in diagnosis is the positive immunohistochemical staining for chromogranin A, synaptophysin, neuron-specific enolase (NSE), and CD56, indicating neuroendocrine differentiation [4,12]. NSE is commonly low in WD-NETs since it indicates tumor aggressiveness, which is not expected in this type of tumor. The Ki-67 proliferation index is usually low (commonly <5%), consistent with their classification as low-grade tumors [4,12]. 

While WD-NETs are generally considered low-grade tumors with indolent behavior as seen in the majority of cases reported in literature and our case, there have been some reports of WD-NETs bladder tumors demonstrating local invasion or distant metastases [6,8,12]. A notable example is the case by Dadhwal et al., which described a patient with a primary bladder WD-NET and concurrent hepatic and peritoneal metastases at diagnosis, emphasizing the need for thorough staging with pathology and imaging even in well-differentiated tumors [8]. 

Due to the extreme rarity of these tumors, no standardized treatment guidelines or follow-up modalities for WD-NETs currently exist due to the limited number of case reports [12]. As a result, therapeutic strategies are often extrapolated from the management of NETs in other organ systems or from general approaches to non-urothelial BCa [7,12]. For localized WD-NETs, transurethral resection alone is often sufficient, as in our case, where repeat cystoscopy showed no evidence of recurrence. Nevertheless, systemic therapy and radical cystectomy may be considered in metastatic or aggressive presentations [8,9]. Other treatment modalities may include somatostatin analogs, chemotherapy, or radiolabeled therapies such as metaiodobenzylguanidine (MIBG), although evidence is limited and based primarily on case reports [8].

## Conclusions

This case report highlights a de novo WD-NET incidentally discovered in a Hispanic adult male following urethroplasty with a buccal mucosal graft for urethral stricture disease. Our patient was asymptomatic, with no LUTS or identifiable risk factors. Diagnosis was basically made on routine cystoscopy surveillance for urethral stricture disease. WD-NETs of the bladder are rare, and their clinical behavior remains poorly characterized due to the limited number of reported cases; however, they are mostly indolent and have a favorable prognosis after surgical resection. This case underscores the importance of considering neuroendocrine pathology even in asymptomatic patients despite their low incidence rate. Finally, due to its limited number of cases reported, this study is a reminder that more research is needed to establish standardized guidelines for the management and long-term follow-up of neuroendocrine bladder tumors.

## References

[1] Le BK, McGarrah P, Paciorek A (2023). Urinary neuroendocrine neoplasms treated in the "modern era": a multicenter retrospective review. Clin Genitourin Cancer.

[2] Koguchi D, Matsumoto K, Shiba I (2022). Diagnostic potential of circulating tumor cells, urinary microRNA, and urinary cell-free DNA for bladder cancer: a review. Int J Mol Sci.

[3] Marletta S, Martignoni G, Ghimenton C, Stefanizzi L, Marcolini L, Caliò A (2023). Well-differentiated neuroendocrine tumor of the urinary bladder expressing GATA 3. Virchows Arch.

[4] Rodriguez Pena MD, Salles DC, Epstein JI (2021). Well-differentiated neuroendocrine tumors of the lower urinary tract: biologic behavior of a rare entity. Hum Pathol.

[5] Chen YB, Epstein JI (2011). Primary carcinoid tumors of the urinary bladder and prostatic urethra: a clinicopathologic study of 6 cases. Am J Surg Pathol.

[6] Humphrey PA, Moch H, Cubilla AL, Ulbright TM, Reuter VE (2016). The 2016 WHO classification of tumours of the urinary system and male genital organs—part B: prostate and bladder tumours. Eur Urol.

[7] Alanee S, Alvarado-Cabrero I, Murugan P (2019). Update of the International Consultation on Urological Diseases on bladder cancer 2018: non-urothelial cancers of the urinary bladder. World J Urol.

[8] Dadhwal R, Jain S, Seth A, Bal CS (2019). Neuroendocrine tumour of urinary bladder: a rare case of aggressively behaving primary well-differentiated neuroendocrine tumour with review of literature. BMJ Case Rep.

[9] Holzbeierlein JM, Bixler BR, Buckley DI (2024). Diagnosis and treatment of non-muscle invasive bladder cancer: AUA/SUO guideline: 2024 amendmen. J Urol.

[10] Coppola A, Gatta T, Pini GM (2023). Neuroendocrine carcinoma of the urinary bladder: CT findings and radiomics signature. J Clin Med.

[11] Oronsky B, Ma PC, Morgensztern D, Carter CA (2017). Nothing but NET: a review of neuroendocrine tumors and carcinomas. Neoplasia.

[12] Shehabeldin AN, Ro JY (2019). Neuroendocrine tumors of genitourinary tract: recent advances. Ann Diagn Pathol.

[13] Thota S, Kistangari G, Daw H, Spiro T (2013). A clinical review of small-cell carcinoma of the urinary bladder. Clin Genitourin Cancer.

